# Cloning and Functional Analysis of Skin Host Defense Peptides from Yakushima Tago’s Brown Frog (*Rana tagoi yakushimensis*) and Development of Serum Endotoxin Detection System

**DOI:** 10.3390/antibiotics13121127

**Published:** 2024-11-24

**Authors:** Taichi Aono, Saki Tamura, Yua Suzuki, Taichi Imanara, Ryosei Niwa, Yoshie Yamane, Tetsuya Kobayashi, Sakae Kikuyama, Itaru Hasunuma, Shawichi Iwamuro

**Affiliations:** 1 Department of Biology, Faculty of Science, Toho University, 2-2-1 Miyama, Funabashi-shi 274-8510, Chiba, Japan; 2 Division of Life Science, Graduate School of Science and Engineering, Saitama University, 255 Shimo-okubo, Sakura-ku, Saitama-shi 338-8570, Saitama, Japan; 3 Department of Biology, Faculty of Education and Integrated Arts and Sciences, Center for Advanced Biomedical Sciences, Waseda University, 2-2 Wakamatsu-cho, Shinjuku-ku 162-8480, Tokyo, Japan

**Keywords:** *Rana tagoi yakushimensis*, frog skin, host defense peptides, yakushimin, amurin-9, brevinin-1, anti-plant pathogenic activity, antioxidant activity, ELEBA, ELGBA

## Abstract

**Background/Objective:** Amphibian skin is a valuable source of host defense peptides (HDPs). This study aimed to identify HDPs with novel amino acid sequences from the skin of *Rana tagoi yakushimensis* and analyze their functions. **Methods:** cDNAs encoding HDP precursors were cloned and sequenced using RT-PCR and 3′-RACE. The novel HDPs were synthesized to evaluate their antimicrobial activity, antioxidant activity, and cytotoxicity. Antimicrobial activity was evaluated by way of broth microdilution and endotoxin- and β-glucan-binding capacity using an enzyme-linked endotoxin binding assay (ELEBA) and a modified ELEBA, respectively. **Results:** Nine cDNAs encoding precursors for various HDP families, including temporin, ranatuerin-2, brevinin-1, amurin-9, and a novel yakushimin peptide, were identified. Brevinin-1TYa exhibited antibacterial activity against *Staphylococcus aureus*, and brevinin-1TYa and amurin-9TYa induced morphological changes in *Escherichia coli* and *S. aureus*. Yakushimin-TYa, amurin-9TYa, and brevinin-1TYa showed concentration-dependent antibacterial effects against the plant pathogens *Xanthomonas oryzae* pv. *oryzae* and *Clavibacter michiganensis* subsp. *michiganensis*. Amurin-9TYa demonstrated strong binding affinity to lipopolysaccharide, lipoteichoic acid, and β-glucan, exhibited antioxidant activity, and lacked cytotoxicity, making it a promising therapeutic candidate. Moreover, brevinin-1TYa showed strong cytotoxicity, whereas yakushimin-TYa exhibited weak cytotoxicity. **Conclusions:** These findings highlight the potential of these peptides, particularly amurin-9TYa, for future applications as antimicrobial and therapeutic agents.

## 1. Introduction

The development of drug-resistant pathogenic microorganisms is a serious public health concern worldwide [1,2,3]. Certain nosocomial pathogens are already resistant to the major available antibiotics. Accordingly, the development of novel antimicrobial agents is anticipated, and host defense peptides (HDPs), also known as antimicrobial peptides, are promising candidates to combat such pathogens [4]. Most HDPs are cationic and hydrophobic and generally adopt an amphipathic α-helical or β-sheet conformation in a membrane-mimetic environment [5,6]. Thus, HDPs are thought to be electrostatically attracted to the negatively charged microbial surfaces and interact with the negatively charged outer leaflet of the microbial cytoplasmic membrane; HDPs can then adopt an amphipathic conformation [7]. In contrast to conventional antibiotics, the antimicrobial activities of HDPs are dependent on their primary and secondary structures, and they can act non-specifically against a wide range of microorganisms [8]. In addition to antimicrobial activity, many HDPs are known to have antifungal, antiviral, endotoxin-binding, chemotactic, and cytokine-expression-modulating effects; therefore, HDPs have been proposed as possible adjuncts or replacements for conventional antibiotics [5,9,10,11].

As amphibians from aquatic habitats adapted to live on land hundreds of millions of years ago, they encountered various pathogenic microorganisms. Unlike fish, reptiles, birds, and mammals, amphibians do not have physical barriers such as scales, shells, feathers, or fur to protect their skin against infections. Therefore, amphibians have developed a strategy to synthesize and secrete diverse HDPs, formerly called antimicrobial peptides, from their skin glands, which are formed during metamorphosis and activated under stress or injury conditions [12,13,14,15]. Moreover, because of their limited adaptive immunity, amphibians depend more on HDP-based innate immunity than other animals. The Ranidae family is one of the most diverse and widely distributed groups of amphibians worldwide, and it has been extensively studied for its HDPs, which exhibit great variation in their amino acid sequences because of their species richness, geographic range, and ecological adaptation [16]. The precursor protein of Ranidae HDPs consists of three regions: a signal peptide region at the N-terminus; an intervening sequence region; and a mature peptide region at the C-terminus. This precursor protein is processed to generate a mature HDP [17]. Ranidae HDPs are classified into different families based on their sequence similarity and are often named after the frog species from which they were first isolated [18]. The signal peptide region is highly conserved among the different genera of Ranidae; the intervening sequence region is conserved among the same families of HDPs; but the mature peptide region is specific to each family [17].

In this study, we focused on the Yakushima Tago’s brown frog (*Rana tagoi yakushimensis*), a distinct subspecies of the Tago’s brown frog (*Rana tagoi*). This particular frog was first identified in the Awa River on Yakushima Island in 1966 [19] and is unique to Japan. There are three Tago’s brown frog subspecies in Japan: the Tago’s brown frog (*R. tagoi*), which is found in Honshu; the Oki Tago’s brown frog (*Rana tagoi okiensis*), which is found on Oki Island in Shimane Prefecture; and the Yakushima Tago’s brown frog (*R. t. yakushimensis*), which is found on Yakushima Island in Kagoshima Prefecture. Previous molecular studies analyzing mitochondrial DNA and genomic DNA from 2012 to 2014 showed that these three subspecies of Tago’s brown frogs and the Stream brown frog, *Rana sakuraii*, their close relative in Japan, could be split into more than a dozen genetic clusters and two major clades [20,21]. The two clades differ in their morphology as follows: the populations from Oki Island and Yakushima Island have been classified as separate subspecies, *R. t. okiensis* and *R. t. yakushimensis*, respectively, based on their morphological features. However, there may be more hidden species in Honshu, the Japanese mainland, according to population ecological and karyological data. We previously isolated skin HDPs from *R. tagoi*, *R. t. okiensis*, and *R. sakuraii* and cloned their precursor cDNAs [14,22,23,24,25]. However, there is no report on HDPs from *R. t. yakushimensis*. In this study, we used total RNA samples extracted from the skin of *R. t. yakushimensis* as a template for the 3′-Rapid Amplification of cDNA End (3′-RACE) method, which used a highly conserved signal peptide region as the forward primer for cDNA amplification. The reverse transcription polymerase chain reaction (RT-PCR) method was also used, which employed a specific sequence of the 3′-untranslated region (3′-UTR) as the reverse primer for cDNA amplification.

Herein, we cloned nine cDNAs encoding precursors for HDPs using this method, compared the amino acid sequences of the obtained HDP precursors among the Tago’s brown frog subspecies and a few Ranid frog species inhabiting the Japanese main island, and performed phylogenetic analysis. Among the *R. t. yakushimensis* HDP precursor cDNAs, we focused on three cDNA clones, which were revealed to encode novel peptide sequences. Furthermore, they were examined for antimicrobial, endotoxin-binding, antioxidant, and cytotoxic properties using synthetic peptide replicates. The three putative novel peptides were named yakushimin-TYa, amurin-9TYa, and brevinin-1TYa. We also modified the previously established enzyme-linked endotoxin binding assay (ELEBA) [26] for the quantitative detection of endotoxins dissolved in fetal bovine serum (FBS) and developed a validation system for the binding of these HDPs to the fungal surface substance β-glucan. The nomenclature used to describe these peptides is the same as that used for other Ranidae skin peptides, where the species is indicated by “TY (*tagoi yakushimensis*)” and isoforms are designated by lowercase letters [18].

## 2. Results

### 2.1. Amplification of HDP Precursor cDNA

**Figure 1:**
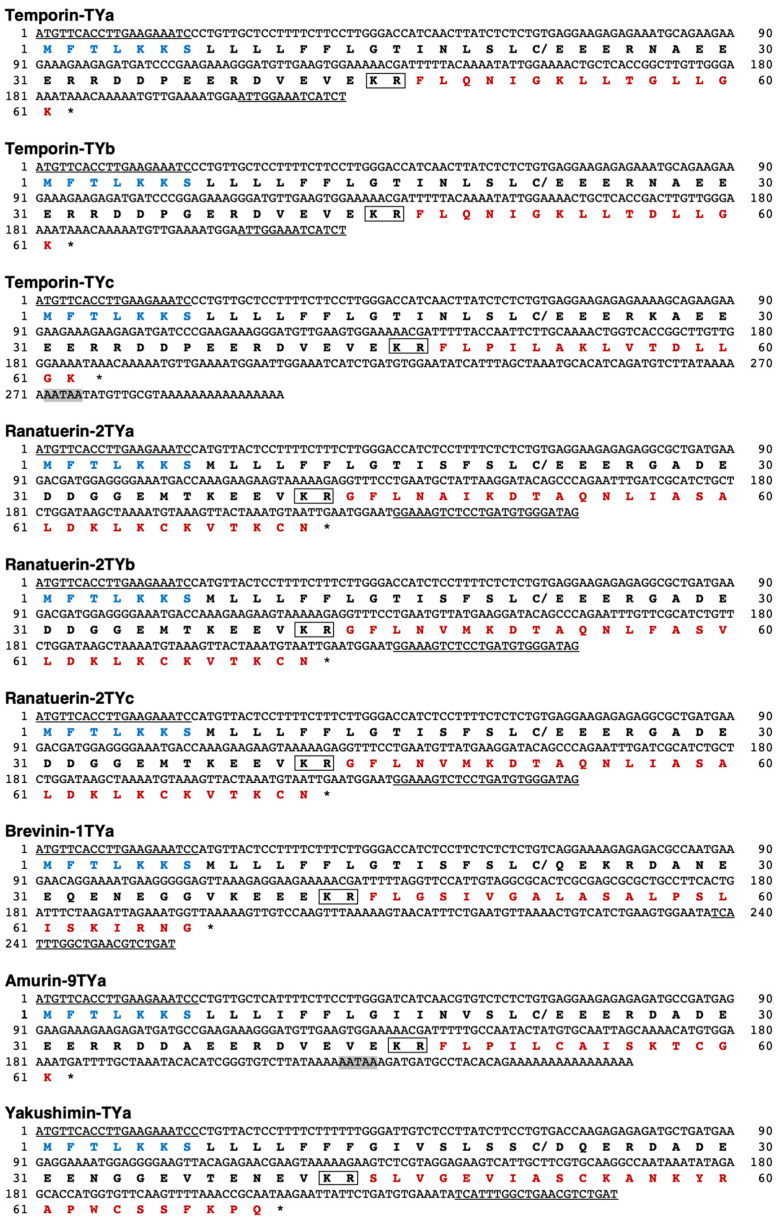
Nucleotide and deduced amino acid sequences of HDP precursor cDNAs that were cloned from *R. t. yakushimensis* skin; in the nucleotide sequences, the forward and reverse primer-derived sequences are underlined. The putative polyadenylation signals are indicated with gray In the amino acid sequences, the forward primer-derived and HDP sequences are indicated with blue and red letters, respectively. Stop codons are marked with asterisks (*), and cleavage sites for signal peptidase and processing enzymes are marked with slashes (/) and boxes, respectively.

We amplified nine cDNA clones encoding HDP precursor proteins using RT-PCR and 3′-RACE, from total RNA extracted from *R. t. yakushimensis* skin (Figure 1). Basic Local Alignment Search Tool (BLAST) analysis revealed that three of the cDNA clones encoded precursors for temporin and ranatuerin-2 family members. For the temporin precursor cDNAs, *R. tagoi* (GenBank Acc. No. AB2194009), *R. t. okiensis* (AB551936), and *R. sakuraii* (AB551935) were at the top of the homology list, while other species such as *Rana ulma* (LC269329, LC269330), *Rana ornativentris* (AB274923), and temporin precursor cDNAs from non-Japanese species such as *Rana boylii* (JQ511851) and *Rana temporaria* (Y09393) also exhibited homology rates greater than 92%. Furthermore, the ranatuerin-2 precursor from *R. ulma* (LC269332) and *R. ornativentris* (AB511966, AB511967) was highly homologous, with homology rates of approximately 84%–90%.

We also obtained cDNA clones encoding the brevinin-1 and amurin-9 families. For the brevinin-1 precursor, several from *R. ulma* (LC269322-269326) were at the top of homology list, all with homology rates > 92%; *R. sakuraii* (AB325526, 325527), *R. t. okiensis* (AB551934, 551934), and the brevinin-1 precursor from *R. japonica* (AB373713) also had rates > 92%. The amurin-9 precursor cDNA was first cloned from the Russian brown frog (*Rana amurensis*) (JF922772), but only the sequence is available, with no reports on the antimicrobial activity or cytotoxicity of the peptide. The remaining clone, when analyzed using BLAST for the intervening peptide sequence (DQERDADEEENGGGEVTENEVKR), showed similarity to odorranaopin (ADP37000) from the Graham’s frog (*Odorrana grahami*) of the family Odorranaidae and ranakinestatin-OS (CDI30156) from the Schmacker’s frog (*Odorrana schmackeri*); however, no known homologous sequence was detected when using only the HDP region for analysis. Therefore, we considered the HDP encoded by this clone as a novel family and named it yakushimin.

The nucleotide sequence information of the nine clones obtained in this study, preprotemporin-TYa (LC848449), preprotemporin-TYb (LC848450), preprotemporin-TYc (LC848451), preproranatuerin-2TYa (LC848452), preproranatuerin-2TYb (LC848453), preproranatuerin-2TYc (LC848454), preprobrevinin-1TYa (LC848455), preproamurin-9TYa (LC848456), and preproyakushimin-TYa (LC848457), has been registered with the GenBank/DDBJ database, and their respective accession numbers are provided in parentheses.

**Figure 2:**
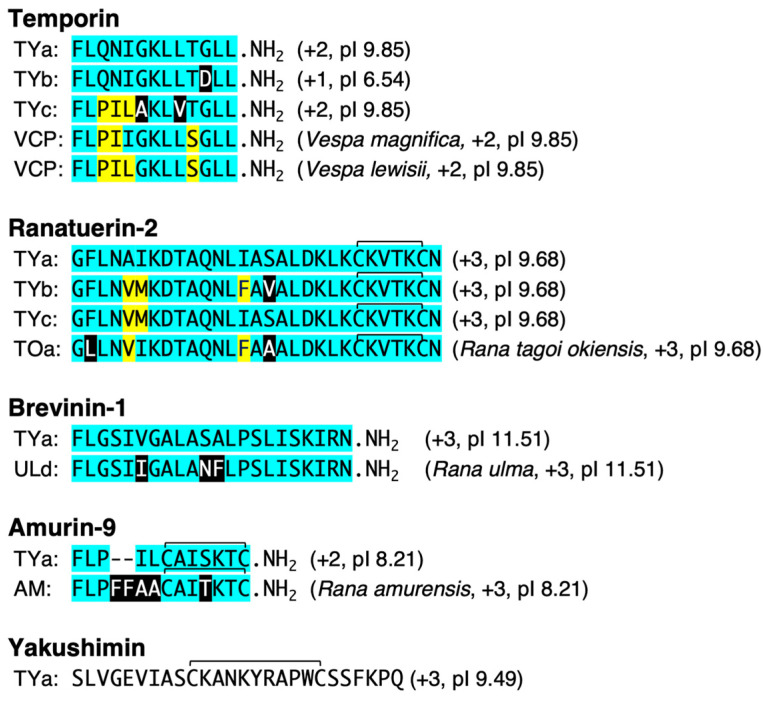
Comparison and schematic alignment of the amino acid sequences of the deduced mature temporin, ranatuerin-2, brevinin-1, amurin-9, and yakushimin peptides, as well as other peptides that exhibited the highest sequence similarities in a BLAST search. Residues that are conserved among each peptide are marked by the same color. The reference sequences of each peptide family are provided at the top of each block; generally, these are the originally isolated sequences. Gaps (-) were introduced to maximize sequence identities. Species names (except for *R. t. yakushimensis* peptides) and the values of the deduced net positive charge and pI are shown in parentheses.

It is known that amphipathic HDPs undergo C-terminal amidation via glycine (Gly)–lysine (Lys) or Gly during processing from the precursor protein, in which Gly becomes the amino group donor [27]. The predicted amino acid sequences of temporin-TY group (a–c) in addition to amurin-9TYa, and brevinine-1TYa, which were obtained from the cDNA sequences in this study, revealed that their C-termini are Gly-Lys and Gly, respectively, which suggests that they are amidated in the mature form (Figure 2). In addition, ranatuerin-2TYa and amurin-9TYa each have two cysteine (Cys) residues, suggesting that they form disulfide bonds in the mature peptides.

### 2.2. Molecular Phylogenetic Tree Analysis

The amino acid sequences of temporin, ranatuerin-2, and brevinin-1 family precursor proteins, including those from *R. t. yakushimensis*, *R. tagoi*, *R. t. okiensis*, *R. sakuraii*, *R. ulma* (endemic to Okinawa Island, Japan), and *Glandirana susurra* (a member of the Ranidae family, endemic to Sado Island, Japan), were previously cloned by us [26,28]. These were compared and molecular phylogenetic tree analysis was performed using the free software MEGA 11 (Figure S1). The results revealed that the amino acid sequences of temporin precursors were diverse both within and among species, while those of ranatuerin-2 were highly conserved within species. The brevinin-1 amino acid sequences were not as diverse as temporin but were not as highly conserved within the same species as ranatuerin-2. Some brevinin-1 precursors additionally displayed homology with temporin precursors, indicating that brevinin-1 and temporin precursors may have diverged from a shared ancestral gene. Phylogenetic relatedness varied among HDP families, and, interestingly, the homology of sequences from the same HDP family may be higher between frogs of different species than between frogs of the same species. Therefore, care should be taken when selecting HDPs for phylogenetic analyses based on the HDP sequence.

### 2.3. Secondary Structure Analysis

BLAST search results revealed that the amino acid sequences of yakushimin-TYa, amurin-9TYa, and brevinin-1TYa were novel. Biochemical characterization of these mature HDPs was performed using Genetyx-Mac version 21.2.0with the Chou–Fasman method and showed that yakushimin-TYa, amurin-9TYa, and brevinin-1TYa had high contents of random coils, β-sheets, and α-helices, respectively, in their secondary structure prediction (Figure S2). Based on these results, synthetic peptides corresponding to each HDP sequence were prepared.

### 2.4. Antimicrobial Assays

**Figure 3:**
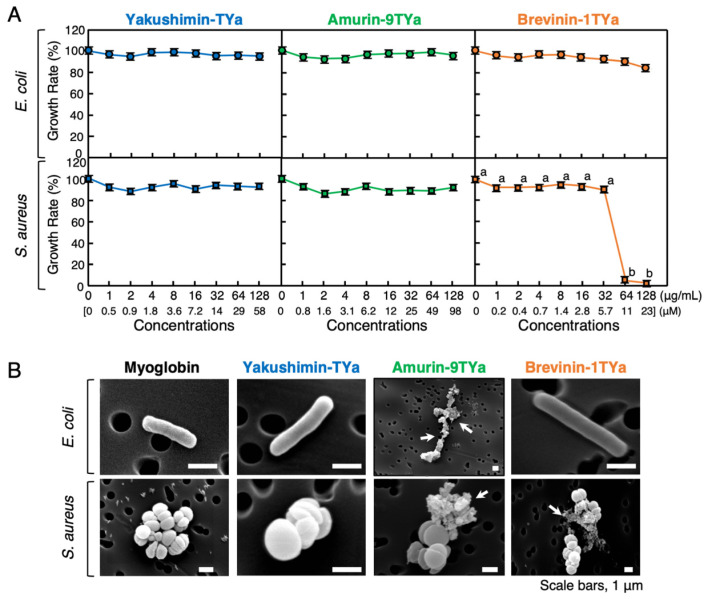
(**A**) Effects of various concentrations of yakushimin-TYa (blue), amurin-9TYa (green), and brevinin-1TYa (orange) on the growth of *Escherichia coli* and *Staphylococcus aureus*. Bacterial cells were incubated with twofold serially diluted peptides for 20 h at 37 °C. Points and vertical bars represent the mean ± standard error of the mean (SE); *n* = 4. Values with the same superscripts are not significantly different (*p* ≥ 0.05). (**B**) Scanning electron microscopy (SEM) analysis of *E. coli* and *S. aureus* cells treated with yakushimin-TYa, amurin-9TYa, or brevinin-1TYa. Bacterial cultures in the mid-log phase were incubated with the various peptides (128 μg/mL) for 1 h at 25 °C and examined by SEM. Abnormally shaped cells indicating cell destruction (arrows) are visible in amurin-9TYa-treated *E. coli* and *S. aureus*, and brevinin-1TYa-treated *S. aureus*, compared to the myoglobin-treated controls. Scale bar = 1 μm.

The broth microdilution method was employed to evaluate the synthetic HDP peptides’ antibacterial activity against pathogenic Gram-negative bacteria, *Escherichia coli*, and Gram-positive bacteria, *Staphylococcus aureus*, in animals. No antibacterial activity was detected against *E. coli* within the concentration range of 0–128 µg/mL for any peptide. However, brevinin-1TYa displayed antibacterial activity against *S. aureus* at concentrations greater than 64 µg/mL (approximately 11 µM), and this was estimated to be the minimum inhibitory concentration (MIC) (Figure 3A). Microstructural observation of bacteria using scanning electron microscopy (SEM) is a common technique used to analyze the mechanism of action of HDPs in terms of their antimicrobial activity. SEM analysis of *E. coli* and *S. aureus* cells treated with 128 µg/mL of each peptide for 1 h revealed that brevinin-1TYa disrupted the cell membrane of *S. aureus* (Figure 3B). Although the broth microdilution method did not detect antimicrobial activity for amurin-9TYa, SEM observation indicated that it caused a disruption to some extent in both *E. coli* and *S. aureus* cells, which suggests weak antimicrobial activity. In contrast, yakushimin-TYa did not induce any morphological abnormalities in either *E. coli* or *S. aureus*.

**Figure 4:**
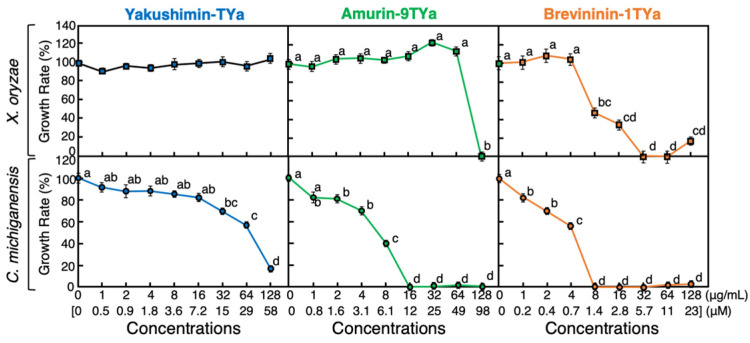
Effects of various concentrations of yakushimin-TYa (blue), amurin-9TYa (green), and brevinin-1TYa (orange) on the growth of plant pathogenic Gram-negative bacteria *Xanthomonas oryzae* pv. *oryzae* and Gram-positive bacteria *Clavibacter michiganensis* subsp. *michiganensis*. Cells of each bacterial strain were incubated with serially diluted yakushimin-TYa, amurin-9TYa, or brevinin-1TYa peptides for 24 h at 28 °C. Points and vertical bars represent the mean ± SE (*n* = 4). In the panels, values with the same superscript are not significantly different (*p* ≥ 0.05).

The antibacterial activity of the three peptides was also evaluated against two plant pathogenic bacteria: *Xanthomonas oryzae* pv. *oryzae* (*X. oryzae*), a Gram-negative bacterium, and *Clavibacter michiganensis* subsp. *michiganensis* (*C. michiganensis*), a Gram-positive bacterium. As shown in Figure 4, yakushimin-TYa showed no significant antibacterial activity against *X. oryzae* in this assay. In contrast, amurin-9TYa inhibited the growth of *X. oryzae* at a concentration of 128 µg/mL (98 µM), while brevinin-1TYa significantly inhibited its growth at concentrations of 8 µg/mL (1.4 µM) and higher. Against *C. michiganensis*, yakushimin-TYa did not completely inhibit bacterial growth even at a concentration of 128 µg/mL (58 µM). However, amurin-9TYa inhibited bacterial growth at concentrations of 16 µg/mL (12 µM) and higher, and brevinin-1TYa inhibited it at concentrations of 8 µg/mL and higher. The MICs of amurin-9TYa and brevinin-1TYa against *X. oryzae* and *C. michiganensis* were calculated to be 98 µM and 5.6 µM, and 12 µM and 1.4 µM, respectively.

### 2.5. Endotoxin Binding Assays

**Figure 5:**
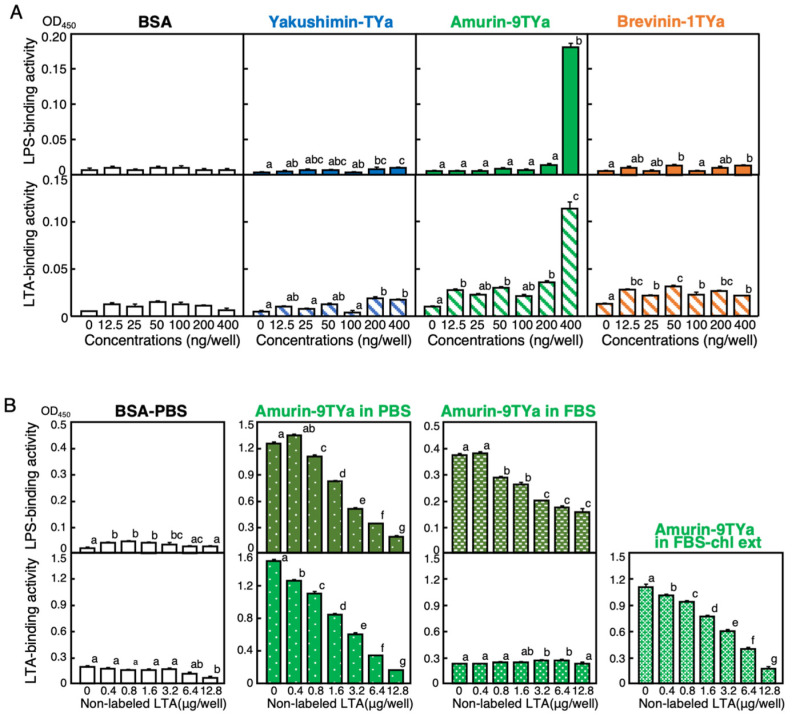
(**A**) Evaluation of the binding abilities of yakushimin-TYa (blue), amurin-9TYa (green), and brevinin-1TYa (orange) to the bacterial endotoxins lipopolysaccharide (LPS) and lipoteichoic acid (LTA) using an enzyme-linked endotoxin binding assay (ELEBA). Twofold serially diluted peptides were coated onto 96-well Immobilizer plates and incubated with biotin-LPS or biotin-LTA for 4 h at 25 °C. Detection was performed using streptavidin-conjugated horseradish peroxidase (SA-HRP) and TMB substrate, and absorbance was measured at 450 nm. (**B**) Assessment of the competitive binding of LPS (dark green) and LTA (light green) to amurin-9TYa and verification in serum. Amurin-9TYa (40 µg/mL) was incubated on Immobilizer plates at 4 °C for 16 h. Biotin-LPS was mixed with LPS in PBS or FBS (500 ng/mL), and biotin-LTA was mixed with non-labeled LTA, both in blocking reagents. For the competition assays with LTA in FBS, a chloroform extraction was performed. The mixture was stirred and centrifuged, and the supernatant was collected, vaporized, and redissolved in endotoxin-free water for the competition experiment. Columns and vertical bars represent the mean ± SE (*n* = 4). Bovine serum albumin (BSA; white) was used as a control. Values with the same letters are not significantly different (*p* ≥ 0.05).

The endotoxin-binding ability of these three peptides was examined using the ELEBA method. The binding activity of amurin-9TYa to the Gram-negative and -positive bacterial surface substances, lipopolysaccharide (LPS) and lipoteichoic acid (LTA), respectively, was detected at 400 ng in a well of 96-well microplates (Figure 5A). It was shown that yakushimin-TYa and brevinin-1TYa had weak binding abilities to LPS and LTA; however, this was statistically significant.

To verify whether labeled and non-labeled endotoxins bind competitively to amurin-9TYa, non-labeled endotoxins at different concentrations were added while keeping the concentrations of amurin-9TYa and labeled endotoxins constant. The results revealed that the amount of non-labeled endotoxins (LPS and LTA) that bound to LPS and LTA, respectively, decreased as the concentration of labeled endotoxins increased. This decrease was linear in both cases, indicating that the binding ability of amurin-9TYa to LPS and LTA is linear in serum. It was then examined whether the binding ability of amurin-9TYa to LPS and LTA remained linear in FBS, i.e., whether it functioned quantitatively. The results revealed that the competitive binding of amurin-9TYa to LPS and LTA was maintained when LPS was dissolved in FBS (Figure 5B). However, the overall absorbance decreased, and the range of competitive quantification was reduced compared to when LTA was dissolved in phosphate-buffered saline (PBS). When LTA was dissolved in serum, no increase in absorbance was observed (Figure 5B). Therefore, a competitive test was performed using “extracted LTA”, which was dissolved and extracted with chloroform, and a competitive decrease in absorbance was observed. This result was lower than the decrease in absorbance observed with extracted FBS without LTA, which indicates that the competition was caused by LTA.

### 2.6. Beta-Glucan Binding Assay

**Figure 6:**
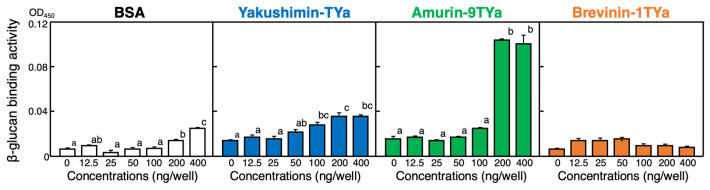
Evaluation of the binding abilities of yakushimin-TYa (blue), amurin-9TYa (green), and brevinin-1TYa (orange) to the fungal cell surface substance β-glucan using a modified ELEBA method, the enzyme-linked β-glucan-binding assay (ELGBA). This method replaces the biotin-labeled endotoxins in the ELEBA method with biotin-labeled β-glucan. The other difference is that the reaction with SA-HRP was extended to 40 min and that with the TMB chromogenic solution to 25 min. Other reaction conditions and details of the preparation of biotin-labeled β-glucan are described in the Section 4. Columns and vertical bars represent the mean ± SE (*n* = 4). BSA (white) was used as a control. In each panel, values with the same letters are not significantly different (*p* ≥ 0.05).

In principle, the ELEBA method can be freely modified in terms of the solid-phase material and the labeled substance bound to it. As a detection system for fungal cell wall components using HDPs has yet to be found, we established the enzyme-linked β-glucan-binding assay (ELGBA) method using β-glucan, a type of fungal surface component, instead of endotoxin, and tested its binding ability to three different peptides. However, biotin-labeled β-glucan is not commercially available. Therefore, we prepared biotin-labeled β-glucan ourselves. As shown in Figure 6, strong binding to β-glucan was observed with amurin-9TYa, as well as weak binding with yakushimin-TYa and no significant binding with brevinin-1TYa. The binding activity trend of these peptides to β-glucan was similar to their binding activity to LPS and LTA.

### 2.7. Antioxidant Assay

**Figure 7:**
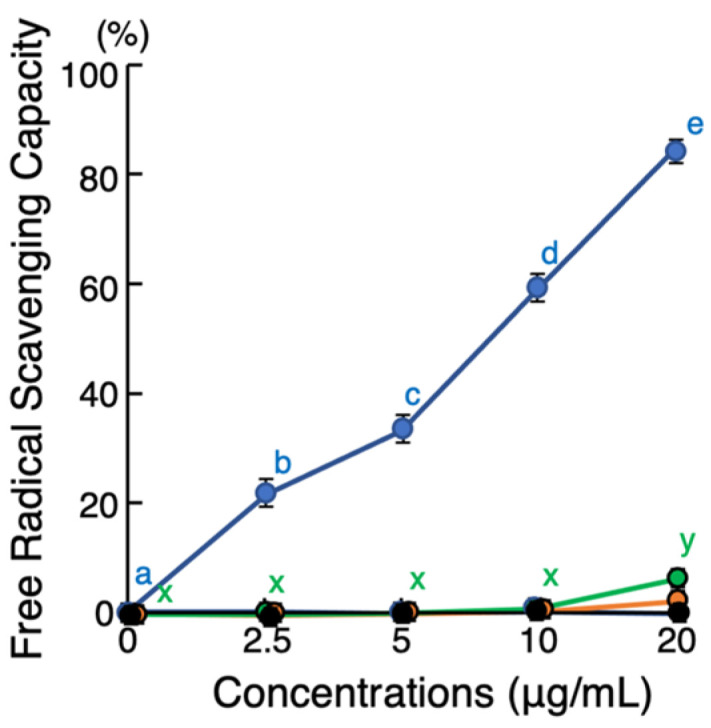
Evaluation of antioxidant activities of yakushimin-TYa (blue), amurin-9TYa (green), and brevinin-1TYa (orange). Twofold serially diluted peptide solutions were incubated with ABTS^+^ in a U-bottom 96-well microplate at 25 °C for 10 min. After incubation, the A_734_ values were measured and the inhibition percentage of ABTS was calculated. Points and vertical bars represent the mean ± SE (*n* = 4). Values with the same letters are not significantly different (*p* ≥ 0.05). Neither brevinin-1TYa nor granuliberin-SSa (black; negative control) [26] exhibited any antioxidant activity.

The antioxidant activities of yakushimin-TYa, amurin-9TYa, and brevinin-1TYa were assessed using the standard 2,2′-azinobis-(3-ethylbenzothiazoline-6-sulfonate) (ABTS) assay (Figure 7). Yakushimin-TYa exhibited strong free radical scavenging activity in a dose-dependent manner, with scavenging capacities of 21%, 34%, 59%, and 85% at concentrations of 2.5, 5, 10, and 20 μg/mL, respectively. Similarly, amurin-9TYa demonstrated antioxidant activity, although to a lesser extent, with a scavenging capacity of 2.6% at 20 μg/mL. In contrast, neither brevinin-1TYa nor granuliberin-SSa (negative control [26]) exhibited any antioxidant activity.

### 2.8. Cytotoxic Assays and Microstructure Observation

**Figure 8:**
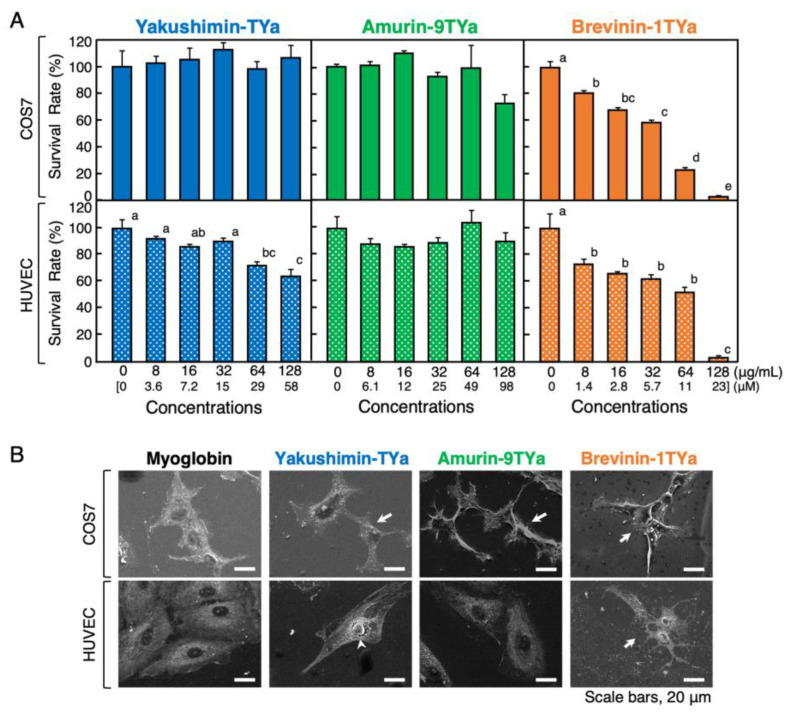
(**A**) Evaluation of cytotoxic effects of yakushimin-TYa (blue), amurin-9TYa (green), and brevinin-1TYa (orange) in eukaryotic cells. Media containing 5 × 10^3^ COS7 cells or 1 × 10⁴ HUVECs in 100 μL aliquots were incubated with twofold serially diluted peptides for 2 h at 37 °C. Cell proliferation was determined using an MTT assay, and the survival rates were expressed relative to the control (0 dose). Columns and vertical bars represent the mean ± SE (*n* = 4). Values with the same superscripts are not significantly different (*p* ≥ 0.05). (**B**) SEM analysis of COS7 cells and HUVECs treated with yakushimin-TYa (128 μg/mL), amurin-9TYa (64 μg/mL), brevinin-1TYa (128 μg/mL), or myoglobin (128 μg/mL, negative control) for 1 h at 25 °C. Abnormally shaped cells, such as those showing cell shrinkage, surface destruction (arrows), or nucleus destruction (arrowhead), are visible in all panels except in the amurin-9TYa-treated HUVECs, compared to the myoglobin-treated cells. Scale bar = 20 μm.

Results from the MTT assay revealed that yakushimin-TYa was weakly cytotoxic to human umbilical vein endothelial cells (HUVECs) (a normal cell line) at a concentration of 128 µg/mL (58 µM), reducing cell viability to 64% (Figure 8A). However, it did not exhibit cytotoxicity against COS7 cells, an African green monkey kidney-derived cell line. Amurin-9TYa was not cytotoxic against either of the cell lines. In contrast, brevinin-1TYa exhibited obvious cytotoxicity, and at the maximum concentration of 128 µg/mL (23 µM), the viability of both cell lines was reduced to less than 2%. The 50% inhibitory concentration (IC_50_) values of brevinin-1TYa against the COS7 and HUVEC cell lines calculated using the least squares method were approximately 49 µg/mL (8.7 µM) and 45 µg/mL (11 µM), respectively. To further investigate the mechanism of HDP cytotoxicity, SEM observations were performed on HDP-treated COS7 cells and HUVECs (Figure 8B). Brevinin-1TYa induced obvious morphological abnormalities in both cell lines compared to the negative control (myoglobin). Although amurin-9TYa did not exhibit significant cytotoxicity against COS7 cells in the MTT assay, morphological abnormalities, such as cell shrinkage, were observed during SEM analysis. Yakushimin-TYa did not appear to cause clear morphological abnormalities in COS7 cells, but it did induce cracks around the nuclei in HUVECs. Regarding the HepG2 cell line, we examined an MTT assay but did not conduct SEM analysis, and significant cytotoxicity was only observed for brevinin-1TYa. Specifically, significant differences in survival rates of 81%, 54%, and 33% were detected at 32, 64, and 128 µg/mL concentrations of brevinin-1TYa treatment, respectively, thus exhibiting concentration-dependent cytotoxicity, with *p* < 0.05 compared to the control.

## 3. Discussion

In this study, cDNAs for nine HDP precursor proteins were obtained from the total RNA of *R. t. yakushimensis* skin using the RT-PCR and 3′-RACE methods. Three of them were cDNA clones of the temporin precursor protein, with a C-terminal amide-type HDP consisting of 13 amino acid residues, which are ubiquitous in many Ranid frog species [11,29,30]. Although all three of the obtained temporin sequences were novel, they were not included in the activity analysis in this study because of their low pI and net positive charge values, thus indicating no expected antimicrobial activity. BLAST analysis showed a higher amino acid sequence homology of temporin-TYc with vespid chemotactic peptide (VCP) than with temporin from other frog species [31]. A peptide homologous to melittin, which is found in honeybee venom, was initially named melittin-related peptide (MRP; GenBank Acc. No. DQ865261); however, this was later reclassified as acyclic brevinin-1 [22]. The reason for the similarity between amphibian and insect peptides, which are not evolutionarily closely related, is unclear. Kambayashi et al. [32] reported horizontal gene transfer from snakes to frogs, but it is unknown whether a similar phenomenon exists between frogs and bees. At the precursor level, there is no amino acid sequence homology between temporin and VCP or brevinin-1 and melittin, and the similarities are limited to the mature peptide region.

Yakushimin-TYa was considered a novel HDP-like family because no HDP family exhibiting homology with the sequence of the HDP coding region was detected in the BLAST search. Amurin-9 cDNA was first cloned from *Rana amurensis*, and the sequence was registered in GenBank (JF922771) as amurin-9AM. The predicted amino acid sequence homology between amurin-9TYa and amurin-9AM was 71% when two gap sequences were inserted, but the activity of amurin-9AM has not been reported. The predicted sequence of the HDP coding region of brevinin-1TYa showed 88% homology with brevinin-1ULd of *R. ulma*, which we have cDNA-cloned, but the biological activity of brevinin-1ULd has also not been verified [28]. Therefore, the bioactivities of yakushimin-TYa, amurin-9TYa, and brevinin-1TYa were evaluated from multiple perspectives in this study.

In the antibacterial activity assay, the broth microdilution method revealed that none of the peptides were active or even weakly active against animal pathogenic bacteria, except for brevinin-1TYa, which inhibited *S. aureus* at high concentrations. SEM analysis revealed that amurin-9TYa caused morphological alterations in *E. coli* and *S. aureus* cells. The peptides also exhibited antimicrobial activity against pathogenic plant bacteria. This phenomenon has also been observed in HDPs from *G. susurra* [26]. This may explain differences in resistance mechanisms between animal and plant pathogenic bacteria. Animal pathogens resist HDPs by altering membrane lipids and LPS structure and producing proteases to degrade HDPs. In contrast, plant pathogens enhance cell wall components and have higher substrate specificity [33]. In addition, animal pathogens mutate rapidly to evade the immune system, while plant pathogens mutate more slowly, thus affecting their resistance to HDPs [34].

Measurements in the ELEBA method are based on the principle of streptavidin binding to endotoxin-labeled biotin [26]. Using this method, a pronounced binding ability to LPS and LTA was detected for amurin-9TYa as well as a modest binding ability for yakushimin-TYa. In the competitive inhibition studies, when peptides bound to endotoxin in the presence of non-labeled endotoxin, increasing the concentration of non-labeled endotoxin decreased biotin-labeled endotoxin binding, which explains the decrease in color development (A_450_). Based on this principle, amurin-9TYa was selected as a solid-phase peptide to quantify LPS and LTA in FBS. Results revealed linearity of the A_450_ values for stepwise decreases in LPS concentration in serum, although sensitivity was reduced. Unlike the Limulus amoebocyte lysate test, which is the conventional LPS quantification method and requires plasma or serum samples and pretreatment because of factors that inhibit the reaction [35], the ELEBA method quantifies serum LPS directly. To detect LTA binding to proteins and peptides conventionally, acid-native polyacrylamide gel electrophoresis (PAGE)-based gel bands for the proteins and peptides have been used for semiquantitative methods that measure the shading [36]. However, there are few reported cases of detection by this method. The competitive ELEBA method revealed A_450_ linearity with a stepwise decrease in LTA concentration, although chloroform extraction pretreatment was required, thus indicating that quantitative analysis is possible for LTA in serum.

In the ELGBA experiments, we initially used curdlan, a typical β-glucan. However, we encountered several problems, including difficulties with water solubility. Therefore, we opted to use Aquaβ, a water-soluble β-glucan that is produced by black yeast (*Aureobasidium pullulans*) and commonly used in food, instead of curdlan. According to the company that markets it, DS WELLFOODS Ltd. (Osaka, Japan), the degree of branching (ratio of β-1,3 to β-1,6 linkages) is at least 90%, which results in high solubility in water (https://sub.osaka-soda.co.jp/wellfoods/beta_glucan/aqua_beta.html, accessed on 10 October 2024). Using Aquaβ, we established a variant of the ELEBA method that can detect the binding of HDP and β-glucan similar to that of LPS and LTA. Detection of β-glucan binding to proteins and peptides was performed using analytical tools and methods, such as surface plasmon resonance [37], isothermal titration calorimetry [38], fluorescence resonance energy transfer [39], and the enzyme-linked immunosorbent assay (ELISA) [40]. The sensitivity and accuracy of the ELGBA method vary greatly depending on the instrument and technique used. In principle, the ELGBA method is very similar to ELISA, but the advantage of ELGBA is that it uses HDP instead of antibodies, thus eliminating the work involved in antibody production and the need to account for differences between antibodies.

Several amphibian HDPs have known antioxidant properties [41,42]. This may be a strategy employed by frog skin, which has no physical barriers such as feathers or scales, to block free radicals that result from UV irradiation. DPPH and ABTS assays are widely used to detect antioxidant activity, and although they often provide similar results, the former is suitable for fat-soluble antioxidants, whereas the latter can detect both water- and fat-soluble antioxidants. As bioprotective peptides contain both hydrophilic and hydrophobic amino acids, the latter is more suitable for measuring their antioxidant activity [43]; thus, the ABTS method was selected for this study. Among the three studied peptides, pronounced antioxidant activity was detected in yakushimin-TYa. The amino acid composition of peptides plays a major role in their antioxidant activity, and the thiol group in Cys is especially important, which is highly reactive with free radicals, as well as the imidazole, phenol, and indole groups in His, Tyr, and Trp, which are also reactive with free radicals [43,44]. Yakushimin-TYa contains two Cys residues and one each of Tyr and Trp; amurin-9TYa has two Cys residues; and brevinin-1TYa lacks all four of these free radical-reactive amino acids. These differences may influence their antioxidant activity.

For the medical application of HDPs, the presence and strength of their cytotoxicity are important, and ideally, they should have high antimicrobial activity and low cytotoxicity. The MTT assay and SEM microstructure analysis revealed that amurin-9TYa and brevinine-1TYa had weak and strong cytotoxicity, respectively, while yakushimin-TYa exhibited no significant activity. Moreover, amurin-9TYa had no antimicrobial activity against animal pathogenic bacteria, but its high endotoxin-binding capacity and antioxidant activity suggest that it is a part of the biological defense system. For example, the application of amurin-9TYa to adsorption therapy in hemodialysis and hemodiafiltration may allow for the removal of endotoxins from blood during sepsis [45,46,47].

## 4. Materials and Methods

All experiments were reviewed and approved by the Toho University Biosafety Committee for Recombinant DNA Experiments (19-54-327), Animal Care and User (18-52-345), and Pathogens (20-53-103, 23-154) and were performed by authorized investigators.

### 4.1. Bacterial and Fungal Cell Strains

The Gram-negative and Gram-positive animal pathogenic bacteria, *E. coli* (JCM5491) and *S. aureus* (JCM2874), respectively, were obtained from the Japan Microbiology Collection (RIKEN BioResource Center, Tsukuba, Japan). The Gram-negative and Gram-positive plant pathogenic bacteria, *X. oryzae* pv. *oryzae* (MAFF311018) and *C. michiganensis* subsp. *michiganensis* (MAFF301254), respectively, were obtained from the National Agriculture and Food Research Organization (Tsukuba, Japan). The aforementioned cell lines were cultivated on suitable agar plates and inoculated into the recommended growth medium, as directed by the supplier, to create a secondary culture for the antimicrobial assay.

### 4.2. Mammalian Cell Lines

COS7 cells, derived from African green monkey kidneys and classified as an immortal cell line, and HepG2 cells, derived from human hepatocellular carcinoma, were obtained from the Health Science Research Resource Bank (Osaka, Japan). HUVECs, a normal human cell line, were obtained from Toyobo (Osaka, Japan). The COS7 and HepG2 cells were cultured in a Dulbecco’s Modified Eagle’s Medium (DMEM) obtained from Nissui (Tokyo, Japan), while the HUVECs were cultured using Lonza’s (Walkersville, MD, USA) EGM-2 BulletKit. All cultures were maintained at 37 °C under 5% CO_2_ and 95% air conditions.

### 4.3. Synthetic Peptides

Putative *R. t. yakushimensis* yakushimin-TYa (SLVGEVIASCKANKYRAPWCSSFKPQ), amurin-9TYa (FLPILCAISKTC.NH_2_), and brevinin-1TYa (FLGSIVGALASALPSLISKIRN.NH_2_) peptides were synthesized by GL Biochem (Shanghai, China) at 87%, 90%, and 89% purity, respectively. A disulfide bond was introduced between Cys^10^ and Cys^20^ in yakushimin-TYa and between Cys^6^ and Cys^12^ in amurin-9TYa. The results of mass spectral analysis of these synthetic peptides were consistent with the theoretical values (Figure S3). These synthetic peptides were diluted to 5 mg/mL as stock solutions in dimethyl sulfoxide and stored at −20 °C.

### 4.4. Amplification of HDP Precursor cDNAs and Molecular Phylogenetic Analysis

Six adult *R. t. yakushimensis* specimens (12–35 mm in body length) were collected on Yakushima Island by an authorized investigator. The frogs were anesthetized with ice-cold water and euthanized via decapitation. The skin was removed, and total RNA was extracted using a modified acid phenol/guanidine isothiocyanate method [26]. HDP precursor cDNA ORFs were amplified using a One-Step RT-PCR Kit (Qiagen, Chatsworth, CA, USA) in 50 µL volumes. A total of 100 ng of RNA was incubated with gene-specific reverse primers and common forward primers at 50 °C for 30 min for reverse transcription, followed by denaturation at 95 °C for 15 min. PCR was performed with initial denaturation at 94 °C for 5 min, followed by 35 cycles of 94 °C for 30 s, 55 °C for 30 s, and 72 °C for 1 min, with a final extension at 72 °C for 7 min. The forward primer 5′-ATGTTCACCATGAAGAAATC-3′ was designed based on the conserved signal peptide region of the Ranid frog HDP precursor [14,23,24,25,26,28]. Three reverse primers (5′-AGATGATTTCCAATTCCAT-3′ for temporin, 5′-CTATCCCACATCAGGAGACTTTCC-3′ for ranatuerin-2, and 5′-ATCAGACGTTCAGCCAAATG-3′ for brevinin-1) were designed according to previous reports [14,23,24,25,26,28] and purchased from Eurofins Genomics (Tokyo, Japan). HDP precursor cDNA was amplified from 200 ng of skin RNA using the 3′-Full RACE Core Set (Takara Bio, Ohtsu, Japan), according to the manufacturer’s instructions. Then, 3′-RACE reactions were conducted with an Oligo dT-3 sites Adaptor Primer Mix and AMV reverse transcriptase (Takara Bio, Ohtsu, Japan) in a 20 μL volume at 30 °C for 10 min, 50 °C for 30 min, 95 °C for 5 min, and 5 °C for 5 min. PCR products were separated on 1.5% agarose gel, purified, and subcloned into the pSTBlue-1 vector using the AccepTor Vector Kit (Novagen, Darmstadt, Germany). Nucleotide sequences were determined via dideoxy chain termination using the Big-Dye Terminator Cycle Sequencing Kit (Applied Biosystems, Foster City, CA, USA) at Eurofins Genomics. Sequence analysis, including identity and predicted secondary structures, was performed using Genetyx-Mac version 21.2.0 (NIHON SERVER Corporation, Tokyo, Japan). The Unweighted Pair Group Method with Arithmetic Mean (UPGMA) with the free software MEGA 11 (https://www.megasoftware.net/, accessed on 25 July 2023) was used to generate the molecular phylogenetic trees between each HDP family ancestor.

### 4.5. Antimicrobial Assay

The antimicrobial activities of serially diluted synthetic peptides against animal pathogenic organisms were determined in 100 μL of cation-adjusted Mueller–Hinton broth (CAMHB) (Becton and Dickinson, Franklin Lakes, NJ, USA) inoculated with log phase cultures (10 μL of 5 × 10^5^ colony-forming units/mL) of the cells of *E. coli* and *S. aureus* in 1% BSA-coated 96-well microtiter cell culture plates at 37 °C in normal air. After incubation for 20 h, the absorbance at 595 nm (A_595_) of each well was measured using an iMark microtiter plate reader (Bio-Rad, Hercules, CA, USA). Similarly, inocula of *X. oryzae* pv. *oryzae* and *C. michiganensis* subsp. *michiganensis* were incubated in CAMHB and Luria–Bertani (LB) broth, respectively, with the peptides at 28 °C for 24 h. Other details are described in our previous study [26,28].

### 4.6. Scanning Electron Microscopy

In the antibacterial experiments, bacterial cells were incubated in 1.5 mL tubes with 400 μL of LB broth until the absorbance at A_595_ reached approximately 0.6. The cells were then incubated with 128 μg/mL of yakushimin-TYa, amurin-9TYa, or brevinin-1TYa for 1 h at 25 °C. After incubation, the cells were collected by gentle centrifugation, prefixed with 2.5% glutaraldehyde for 1 h, fixed with 1% OsO_4_ for another hour, and washed with phosphate buffer (PB). Dehydration was performed using a series of ethanol concentrations (50%, 70%, 90%, 95%, and 100%) with a nano-percolator filter (JEOL, Tokyo, Japan). The cells were then incubated with a 1:1 mixture of ethanol and t-butyl alcohol for 1 h, followed by incubation with 100% t-butyl alcohol for 20 min, repeated three times. Samples were frozen at 4 °C, lyophilized, and coated with 15 nm gold particles using the Quick Coater SC701 (Sanyu Electron, Tokyo, Japan). Morphological observations were performed using a JEOL JSM-6390LV scanning electron microscope (SEM). For cytotoxicity experiments, COS7 cells and HUVECs were precultured on 12 mm poly-l-lysine-coated coverslips (Corning, New York, NY, USA) placed in 24-well cell culture plates. The cells were then incubated with *R. t. yakushimensis* HDP under the same conditions as those outlined in Section 4.9. After incubation, the SEM samples were prepared as previously described and subjected to SEM analysis per our previous reports [26,28].

### 4.7. Endotoxin- and β-Glucan-Binding Assays

#### 4.7.1. Enzyme-Linked Endotoxin Binding Assay (ELEBA)

To evaluate the binding abilities of yakushimin-TYa, amurin-9TYa, and brevinin-1TYa to bacterial cell surface substances, LPS and LTA, we performed ELEBA according to the method detailed in our previous study [26]. Briefly, 100 μL solutions of serially diluted yakushimin-TYa, amurin-9TYa, and brevinin-1TYa (0–4 mg/mL) were coupled in 100 mM carbonate buffer (pH 9.6) within a well of a 96-well Nunc Immobilizer Amino microtiter plate (Thermo Scientific, Waltham, MA, USA). After overnight incubation at 4 °C, the wells underwent post-coupling treatment with 10 mM ethanolamine in the carbonate buffer. Subsequently, they were washed with PBS (pH 7.2) containing 0.05% (*w*/*v*) Tween 20 (PBS-T). Following incubation with an ELISA blocking reagent (Sigma-Aldrich, St. Louis, MO, USA) for 1 h at 25 °C, the wells were washed with PBS-T and incubated with 100 μL of 500 ng/mL biotinylated LPS (biotin-LPS; InvivoGen, San Diego, CA, USA) prepared from *E. coli* O111:B4 or biotinylated LTA (biotin-LTA) prepared from *S. aureus* (InvivoGen) [26] in 100 μL of the blocking reagent for 2 h at 25 °C. Following the blocking procedure, the wells were washed with PBS-T and incubated with 100 μL of streptavidin-conjugated horseradish peroxidase (SA-HRP; PerkinElmer, Waltham, MA, USA) diluted with the blocking reagent at 25 °C for 30 min. The liquid in the wells was then aspirated, and the wells were washed with PBS-T and PBS and then reacted with 100 μL of the substrate solution from the ELISA POD substrate TMB kit (Nacalai Tesque, Kyoto, Japan) for 15 min at 25 °C in the dark. The reaction was terminated by adding 100 μL of 1 M H_2_SO_4_. The absorbance at 450 nm was measured using a microtiter plate reader.

#### 4.7.2. Competitive ELEBA

Based on the results of the ELEBA experiment described above, clear binding capabilities of amurin-9TYa with LPS and LTA were detected. To verify whether this binding was specific, we attempted to establish a competitive ELEBA method by advancing the ELEBA technique. One hundred aliquots of amurin-9TYa (40 µg/mL) were added to wells of the Immobilizer plate and incubated at 4 °C for 16 h for solidification. Biotin-LPS was mixed with LPS in PBS or FBS dissolved in the blocking reagent (final concentration of 500 ng/mL). Similarly, non-labeled LTA was serially diluted in endotoxin-free water and mixed with biotin-LTA diluted 5000-fold in the blocking solution. Non-labeled LTA of different concentrations, also diluted in endotoxin-free water, was added to each well (100 µL per well). For competition with the LTA solution dissolved in FBS, an equal volume of chloroform was added to the FBS solution of LTA. The mixture was stirred for 2 h and centrifuged at 4 °C for 10 min at 10,000 rpm, and the supernatant was collected. After vaporization, the supernatant was redissolved in endotoxin-free water and used for the competition experiment, following the same procedure described above.

#### 4.7.3. Enzyme-Linked β-Glucan Binding Assay (ELGBA)

To evaluate the binding abilities of the peptides to β-glucan, a fungal cell surface substance, we modified the ELEBA and developed an ELGBA. In this study, we selected Aquaβ, a β-1,3 glucan with a high branching ratio of side-chain glucose (almost 100% branching), from *A. pullulans* sourced from DS WELLFOODS (Osaka, Japan) as a β-glucan specimen. Biotinylation of Aquaβ was performed using the Dojindo online protocol (https://www.dojindo.co.jp/technical/protocol/p45.pdf, accessed on 23 February 2023). Briefly, Aquaβ was suspended in MES buffer (10 mM MES, Dojindo, Kumamoto, Japan; 15 mM NaCl; pH 5.5) at a concentration of 50 mM. Subsequently, NaOH was added and heated to 80 °C for 1 h to achieve complete dissolution, followed by the addition of HCl to adjust the pH to 6.0. The resulting solution was concentrated using an Amicon Ultra 0.5 mL Centrifugal Filter Ultracel-10K (Merck Millipore, Burlington, MA, USA) at 4 °C for 15 min at 14,000× *g*. After collecting the centrifugation product, 1/250 volume of NaIO_4_ (Wako, Osaka, Japan) dissolved in MES buffer was added and allowed to stand on ice for 20 min to introduce an aldehyde into the β-1,3 glucan. Excess NaIO_4_ was removed by ultrafiltration, and the aldehyde product was diluted with MES buffer to approximately 400 μL. Finally, 4 μL of N″-Biotinyl-6-aminohexanoylhydrazide (TCI Chemicals, Tokyo, Japan) solution (185 mg/mL DMSO) was added. The mixture was shaken in a thermostatic bath at 25 °C for 4 h. After shaking, the final product was collected by ultrafiltration, which served as the biotinylated β-glucan (bio-β-glucan), and was stored at 4 °C. Subsequent experimental procedures were performed following the described ELEBA method. Biotinylated β-glucan was diluted 100-fold at the time of use and 100 μL per well was added. However, the reaction time with SA-HRP was extended to 40 min, and the coloration time with the TMB chromogenic solution was extended to 25 min.

### 4.8. Antioxidant Assay

Antioxidant activity was measured using the ABTS cation radical scavenging assay. To prepare the stock solution, 2.5 mL of 14 mM ABTS (Sigma-Aldrich) and 88 μL of 140 mM potassium persulfate were combined and incubated in the dark at 25 °C for 16 h. An 18 μL aliquot of the working solution was incubated with 20 μL of yakushimin-TYa, amurin-9TYa, or brevinin-1TYa at final concentrations of 0, 5, 10, or 20 μg/mL in the wells of a U-bottom 96-well microplate at 25 °C for 10 min. The A_734_ values of the samples were then measured, and the percentage inhibition of ABTS was calculated using the following formula:

ABTS cation radical scavenging activity (%) = [(A_blank_ − A_sample_)/A_blank_] × 100

### 4.9. Cytotoxicity Assay

To assess the cytotoxic effects of the *R. t. yakushimensis* HDPs on eukaryotic cells, standard MTT assays were performed according to the method described in our previous study [26,28]. Briefly, 5 × 10^3^ COS7 cells or 1 × 10^4^ HUVECs were cultured in the wells of 96-well microtiter cell culture plates (AS ONE, Osaka, Japan) containing 100 μL of the appropriate media, described in Section 2.2, at 37 °C overnight in a 5% CO_2_ atmosphere. The media were replaced with fresh media containing yakushimin-TYa, amurin-9TYa, or brevinin-1TYa at final concentrations of 0, 32, 64, and 128 μg/mL, and the cells were incubated for an additional 24 h. Thereafter, 100 μL of lysis buffer combined with 6 N HCl and isopropanol (0.34/99.66, *v*/*v*) was added, and the cells were cultured overnight. The A_570_ values of the samples were measured, and the survival rate was calculated based on the reduction in MTT values relative to those of the control cells (0 dose in 24 h culture). Moreover, 1% (*v*/*v*) Triton X-100 was used as a positive control, which reliably inhibited cell growth in the cell viability test. After incubation, SEM samples were prepared and analyzed as previously described [26,28].

### 4.10. Statistical Analysis

Statistical analyses of data from the antimicrobial assay, ELEBA, ELGBA, free radical scavenging assay, and cytotoxic assay were performed using an analysis of variance followed by multiple comparisons using Scheffé’s *F*-test. Differences were considered significant at *p* < 0.05.

## 5. Conclusions

We cloned nine cDNAs encoding biosynthetic precursors for the temporin, ranatuerin-2, brevinin-1, and amurin-9 families, along with a novel peptide from the yakushimin family, from RNA of *R. t. yakushimensis* frog skin. Among the tested peptides, brevinin-1TYa exhibited antibacterial activity against *S. aureus*, while SEM analysis revealed that both brevinin-1TYa and amurin-9TYa induced morphological changes in *E. coli* and *S. aureus*. Furthermore, yakushimin-TYa, amurin-9TYa, and brevinin-1TYa exhibited concentration-dependent antibacterial effects against the plant pathogens *X. oryzae* and/or *C. michiganensis*. Notably, amurin-9TYa displayed a strong binding affinity to LPS, LTA, and β-glucan, while yakushimin-TYa and brevinin-1TYa showed weaker interactions. Amurin-9TYa also demonstrated antioxidant activity and lacked cytotoxicity, making it a promising candidate for future therapeutic development. In contrast, brevinin-1TYa exhibited relatively strong cytotoxicity, and yakushimin-TYa showed only weak cytotoxicity. These findings emphasize the potential of these peptides, particularly amurin-9TYa, for future applications as antimicrobial and therapeutic agents.

## Data Availability

The original contributions presented in this study are included in the article/Supplementary Material. Further inquiries can be directed to the corresponding author.

## Supplementary Materials

The following supporting information can be downloaded at https://www.mdpi.com/article/10.3390/antibiotics13121127/s1. Figure S1: Molecular phylogenetic tree of the HDP precursor proteins of *R. t. yakushimensis* and its subspecies and relatives, as well as other Ranid frog species; Figure S2: Deduced amino acid sequences and helical wheel projections of the mature *R. t. yakushimensis* peptides. Figure S3: Results of purity and mass analyses of the generated synthetic peptides by high-performance liquid chromatography and mass spectrometry.
