# Supplementary material for: Cloning and Functional Analysis of Skin Host Defense Peptides from Yakushima Tago’s Brown Frog (Rana tagoi yakushimensis) and Development of Serum Endotoxin Detection System
Source: Antibiotics (Basel). 2024 Nov 24;13(12):1127. doi: 10.3390/antibiotics13121127 (PMC11672578; doi:10.3390/antibiotics13121127)
Supplement: Supplementary file 1 [file antibiotics-13-01127-s001.zip › antibiotics-3314446-supplementary.pdf]

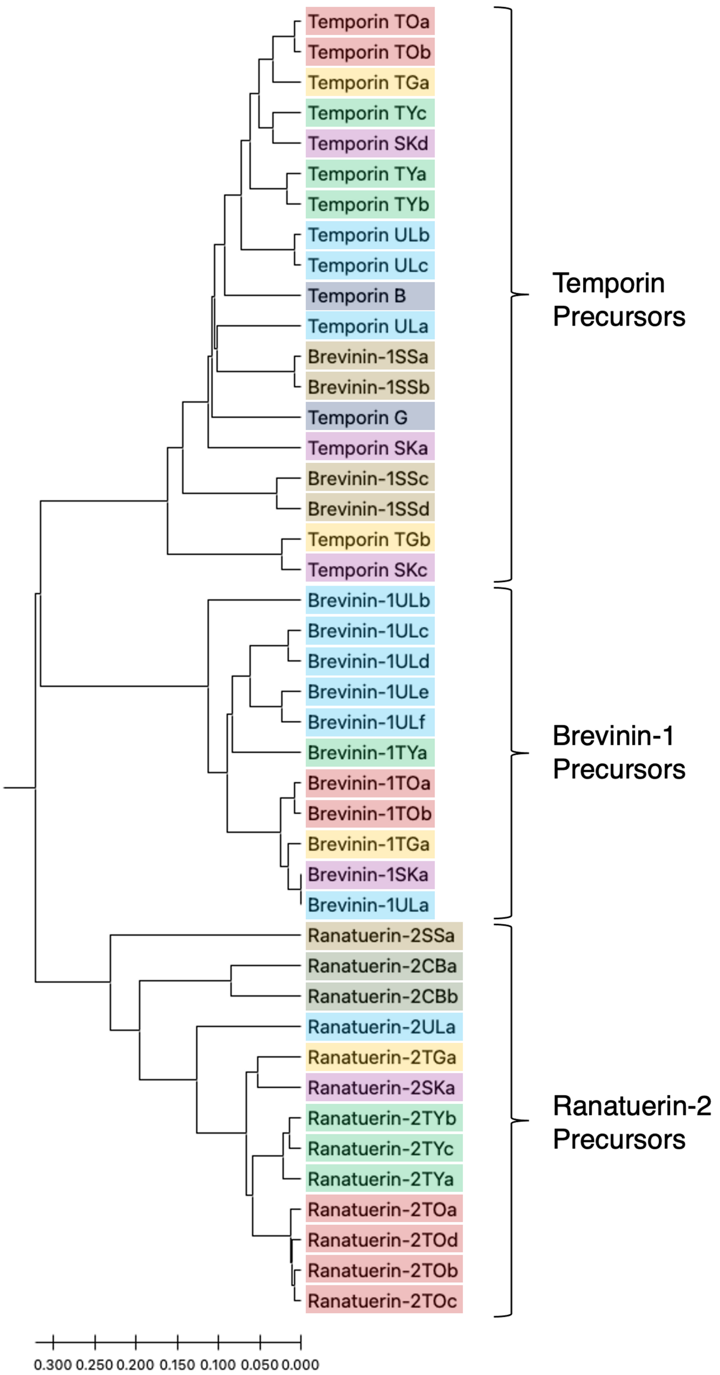

Figure S1. Molecular phylogenetic analysis of host defense peptide (HDP) precursor proteins was performed using the Unweighted Pair Group Method with Arithmetic Mean method. Amino acid sequences of temporin, ranatuerin-2, and brevinin-1 precursors from *Rana tagoi yakushimensis* (TY), *Rana tagoi* (TG), *Rana tagoi okiensis* (TO), *Rana sakuraii* (SK), *Rana ulma* (UL), *Rana temporaria* (B and G), *Lithobates catesbeianus* (CB), and *Glandirana susura* (SS) were analyzed. A phylogenetic tree was constructed using MEGA11. The HDPs from the same species are marked with the same color, indicating their high sequence homology. Among temporin precursors, a significant sequence diversity was observed, whereas high interspecies homology was observed among ranatuerin-2 precursors. Brevinin-1 precursors also exhibit significant interspecific homology, and some additionally display homology with temporin precursors, indicating that brevinin-1 and temporin precursors may have diverged from a shared ancestral gene. The scale bar in the figure indicates the rate of amino acid substitution.

Yakushimin-TYa

Sequence: SLVGEVIASCKANKYRAPWCSSFKPQ

Secondary structure: HHHHHBBBBBHHHHCCCCCCCCCCCC

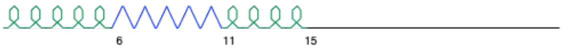

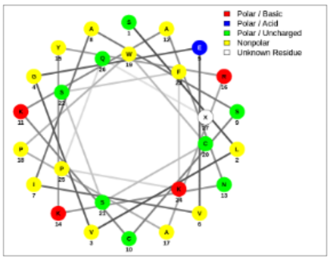

Molecular weight	2868.29
Isoelectric point	9.49
Net positive charge	+3
Hydrophobic amino acid	12 (46.15%)
Neutral amino acid	8 (30.77%)
Hydrophilic amino acid	6 (23.08%)

Amurin-9TYa

Sequence: FLPILCAISKTC.NH_2

Secondary structure: BBBBBBBBBCCC

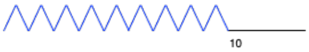

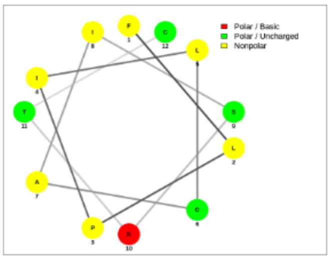

Molecular weight	1305.65
Isoelectric point	12.99
Net positive charge	+2
Hydrophobic amino acid	7 (58.33%)
Neutral amino acid	4 (33.33%)
Hydrophilic amino acid	1 (8.33%)

Brevinin-1TYa

Sequence: FLGSIVGALASALPSLISKIRN.NH_2

Secondary structure: HHHHBBBBBHHHHHHHHHHHHC

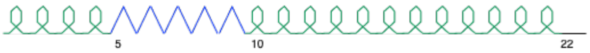

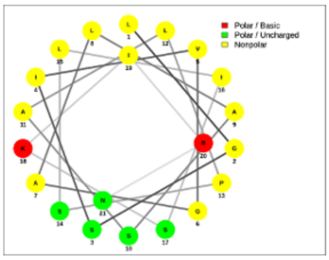

Molecular weight	2868.29
Isoelectric point	12.99
Net positive charge	+3
Hydrophobic amino acid	15 (68.18%)
Neutral amino acid	5 (22.73%)
Hydrophilic amino acid	2 (9.09%)

Figure S2. Amino acid sequences and compositions were used to calculate the molecular weight, isoelectric points, net charges, and predicted secondary structures and the helical wheel projections of yakushimin-TYa, amurin-9TYa, and brevinine-1TYa. In the secondary structure, H, B, and C indicate an α-helix, β-turn, and random coil, respectively. In the helical wheel projections, polar/basic, polar/acidic, polar/uncharged, and nonpolar amino acids are marked in red, blue, green, and yellow, respectively. The predicted secondary structures of yakushimin-TYa and amurin-9TYa do not reflect the effect of disulfide bonds because of the nature of the analysis software.

Yakushimin-TYa

HPLC

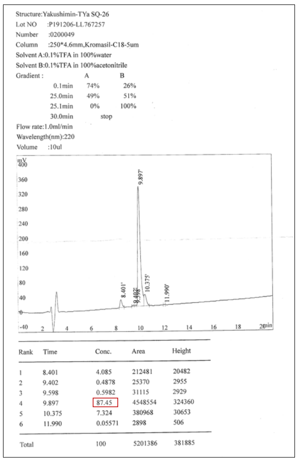

Mass spectrum

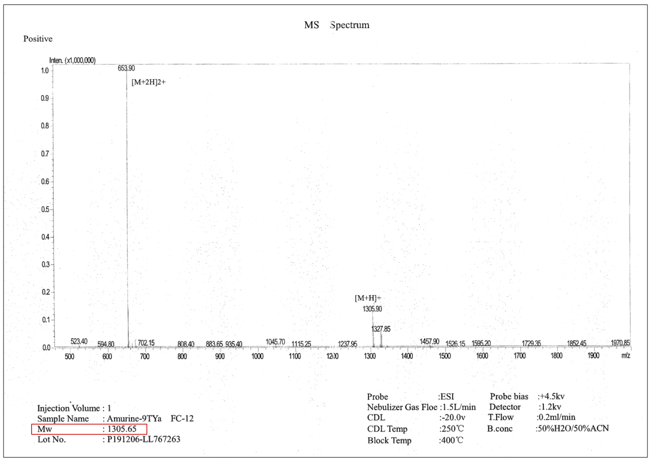

Amurin-9TYa

HPLC

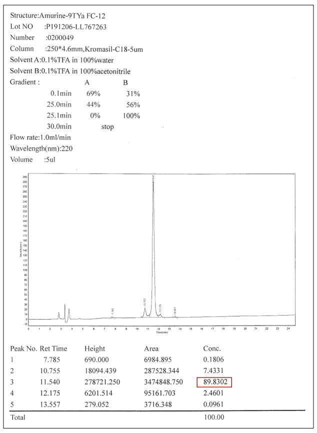

Mass Spectrum

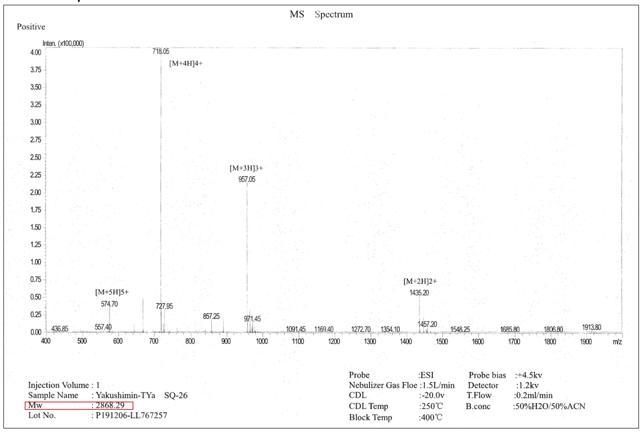

Brevinin-1TYa

HPLC

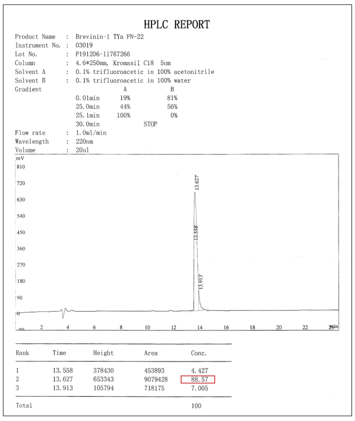

Mass spectrum

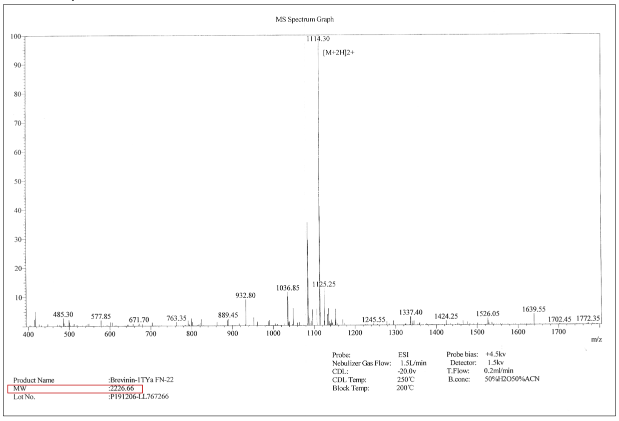

Figure S3. Results of purity and mass analysis of the generated synthetic peptides (yakushimin-TYa, amurin-9TYa, and brevinin-1TYa) by high-performance liquid chromatography (HPLC) and mass spectrometry. These charts and graphs were provided by the company that synthesized the peptides (GL Biochem) and are accompanied by a letter of quality assurance.
